# Supplementary material for: Yiai Fuzheng decoction inhibits triple-negative breast cancer by remodeling the immune microenvironment
Source: Front Immunol. 2025 Sep 30;16:1615631. doi: 10.3389/fimmu.2025.1615631 (PMC12518410; doi:10.3389/fimmu.2025.1615631)
Supplement: Supplementary file 2 [file DataSheet1.zip › Supplementary File 2/Treatment/C_vs_H/07_Pathway_Analysis/Pathway_Analysis/Sig_Pathways_ID/Pentose and glucuronate interconversions.pdf]

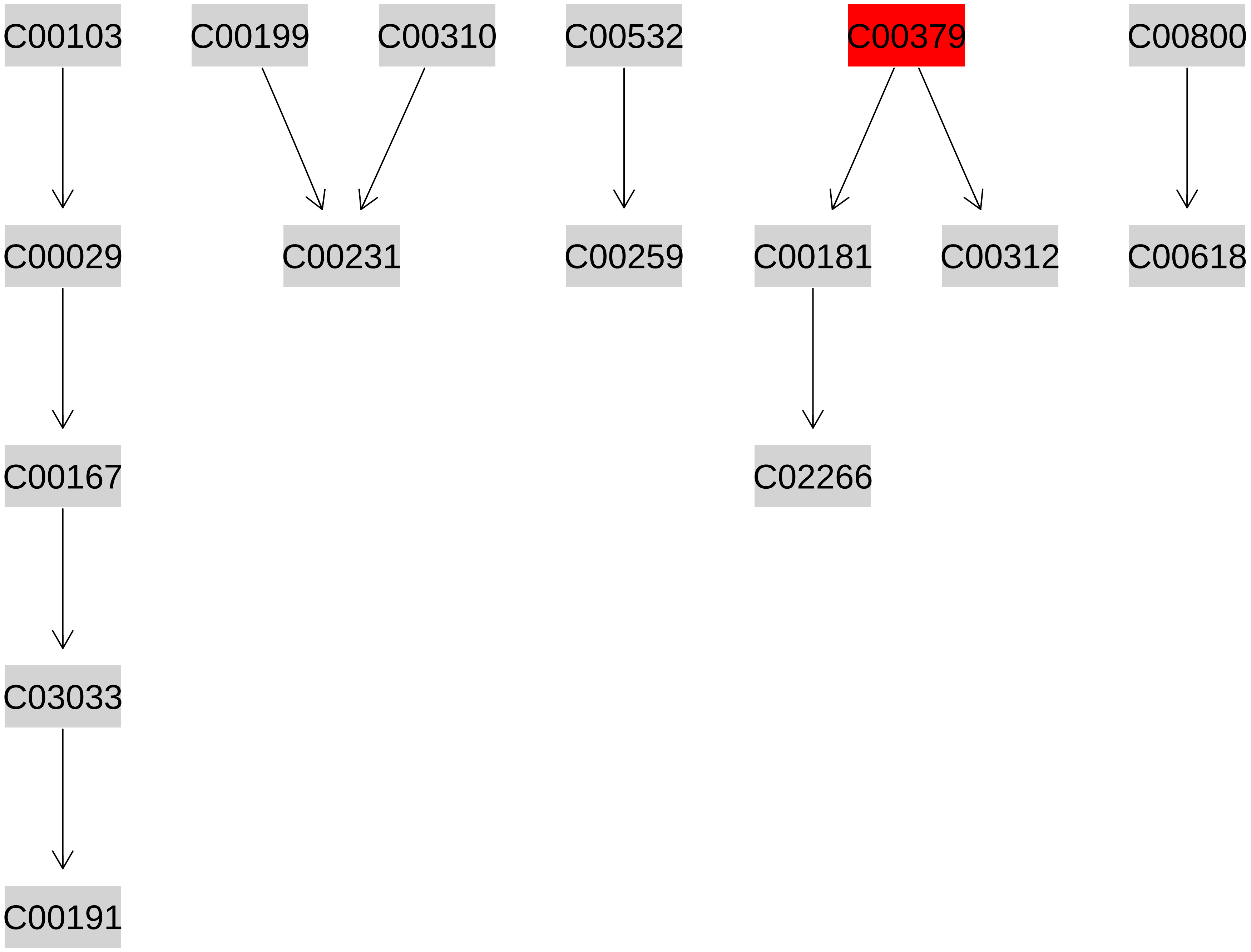
C00103
C00199
C00310
C00532
C00379
C00800
C00029
C00231
C00259
C00181
C00312
C00618
C00167
C02266
C03033
C00191
